# Supplementary material for: Empagliflozin Protects HK-2 Cells from High Glucose-Mediated Injuries via a Mitochondrial Mechanism
Source: Cells. 2019 Sep 14;8(9):1085. doi: 10.3390/cells8091085 (PMC6770192; doi:10.3390/cells8091085)
Supplement: Supplementary file 1 [file cells-08-01085-s001.pdf]

Supplementary Materials

Measurement of intracellular glucose levels

HK-2 cells were treated without/with empagliflozin at the concentration of 100 and 500 nM for 24 h. After that, glucose uptake was assessed using a Glucose Uptake Assay Kit (ab136955, Abcam, Cambridge, UK) following manufacturer's instructions. Briefly, cells were starved in serum free medium overnight and then Krebs–Ringer–Phosphate–Hepesbuffer with 2% BSA for 20 min. After insulin stimulation, the glucose analog 2-Deoxyglucose (2-DG) was added to cells and the accumulated 2-DG6P was oxidized to generate NADPH, which resulted in oxidation of a substrate. The oxidized substrate then can be detected at OD = 412 nm.

RNA interference

RNA interference experiments were performed with appropriate controls. Small interfering RNA (siRNA) oligonucleotides were purchased from Santa Cruz Biotechnology (Santa Cruz, USA). siRNA transfections were performed in a 12-well plate. Each treatment was performed in triplicate. Cells were plated at a density of 8×10^4 cells/well in plating medium overnight. The medium was replaced with fresh plating medium 30 min before transfection. For each well, 1 μL of siRNA and 3 μL of Metafectene Pro (Biontex, Germany) were diluted in 100 μL of serum/antibiotic-free medium in two separate tubes and incubated at room temperature for 5 min. Transfection mix was made by mixing the solutions of these two tubes and incubated for 30 min at room temperature. Subsequently, 800 μL of transfection mix was added dropwise into each well to generate 20 nM siRNA. After gentle rocking, the plate was placed in an incubator with 5% CO2 at 37°C.

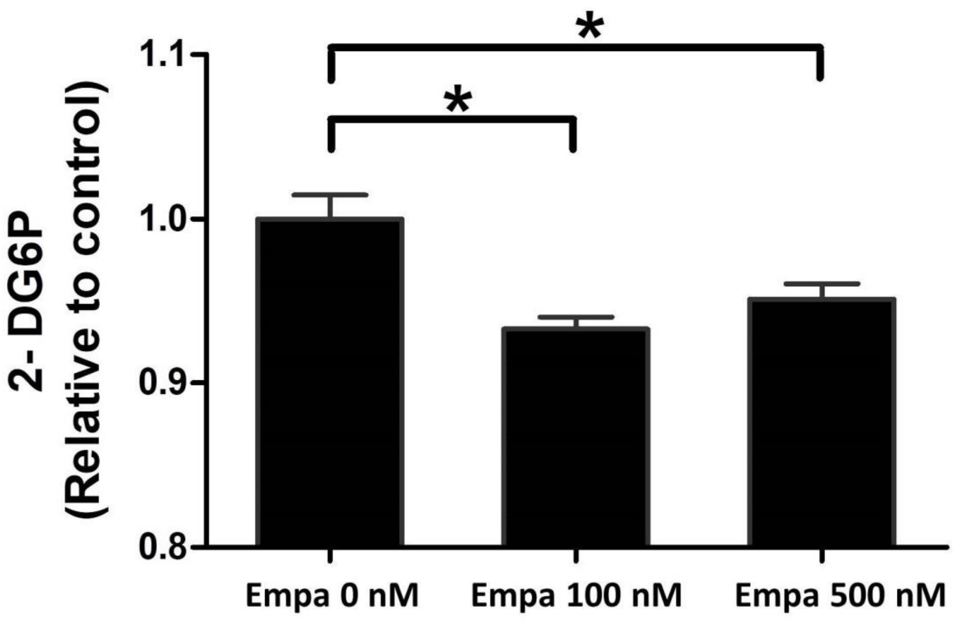

Figure S1. Empagliflozin reduces intracellular glucose. Intracellular glucose levels were measured by glucose uptake assay. Data are expressed as mean ± SEM. *$P < 0.05$. Empa, empagliflozin.

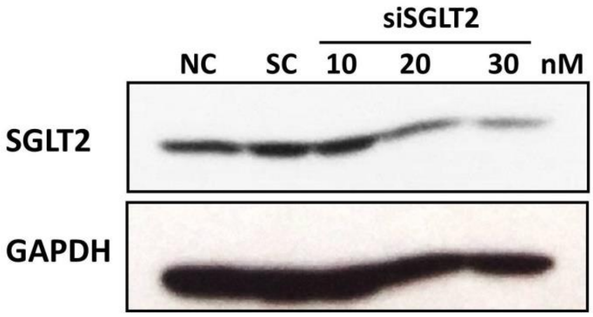

Figure S2. SGLT2 knocked down by siSGLT2. In HK-2 cells, SGLT2 was effectively knocked down by siSGLT2 at the concentration of 20 and 30 nM. NC, negative control; SC, scrambled siRNA

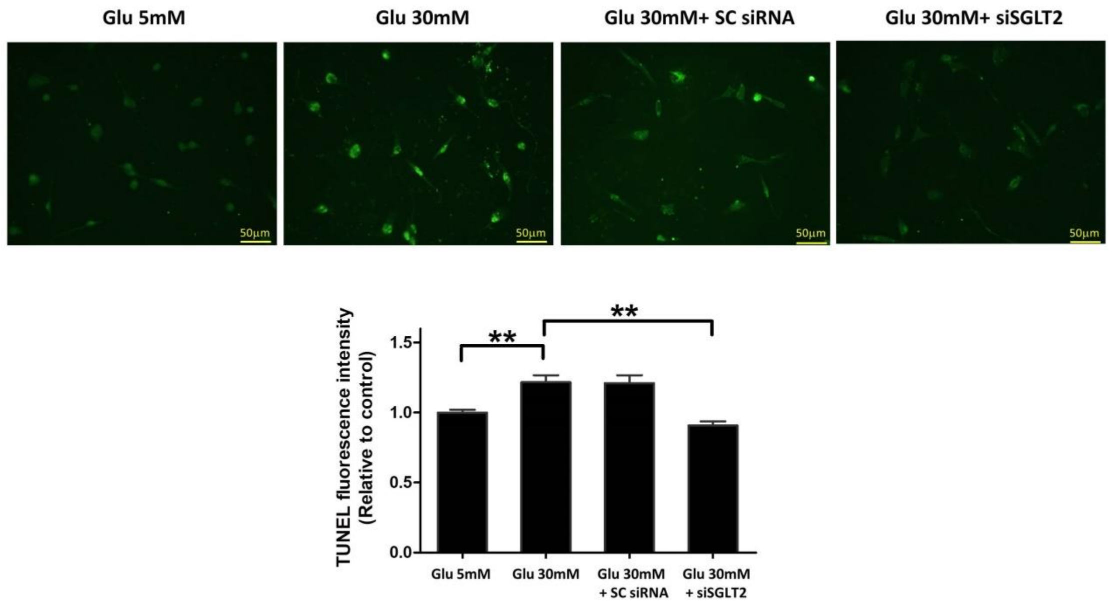

Figure S3. Silencing of *SGLT2* reduces the high glucose-induced apoptosis of HK-2 cells. Fluorescence images show positive TUNEL staining in the four treatment groups. Quantitative analysis of TUNEL assay shows that high glucose induces apoptosis and siSGLT2 ameliorates this effect. Data are expressed as mean ± SEM. $^{**}P < 0.001$. siSGLT2, small interfering RNA targeting *SGLT2*; SC, scrambled.

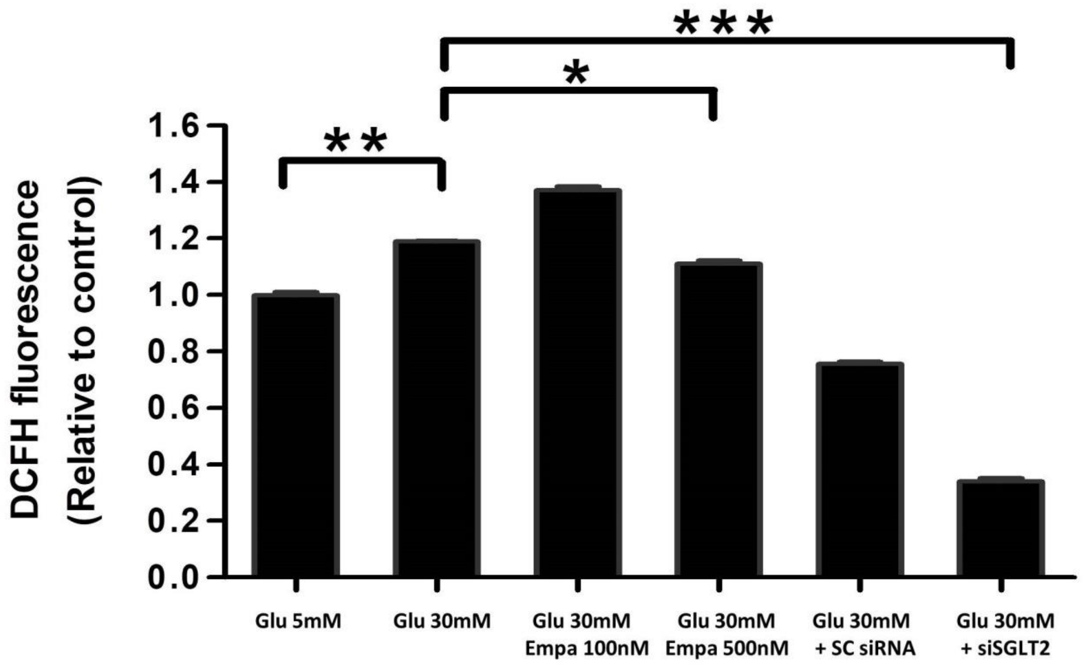

Figure S4. Empagliflozin and siSGLT2 reduce high glucose-induced cellular ROS production in HK-2 cells. Data were obtained from three independent experiments and are expressed as mean ± SEM. $^{*}P < 0.05$, $^{**}P < 0.001$, $^{***}P < 0.0001$. siSGLT2, small interfering RNA targeting *SGLT2*; SC, scrambled.
